# Supplementary material for: Are carbon emissions trading and green financial instruments synergistic? -Comprehensive quantitative research based on content analysis
Source: PLoS One. 2024 Mar 7;19(3):e0298601. doi: 10.1371/journal.pone.0298601 (PMC10919601; doi:10.1371/journal.pone.0298601)
Supplement: S1 Data — (ZIP) [file pone.0298601.s001.zip › Data/地级市Z_F工作报告.zip!/╜¡╬≈/╨┬╙α╩╨/└┤╫╘╣1⁄2╓┌║┼í╛┬φ┐╦╩2╛▌═°í┐_2004.docx]

各位代表：

现在，我代表市人民政府向大会作政府工作报告，请予审议，并请市政协委员和其他列席会议的同志提出意见。

一、过去一年经济社会的快速发展，实现了率先建设全面小康社会的良好开端

２００３年，是本届政府工作的第一年，也是全面建设小康社会的起步之年。一年来，全市上下深入贯彻党的十六大和十六届三中全会精神，以“江西崛起，新余率先”为主题，紧紧围绕“一条主线，五篇文章”的总体思路，与时俱进，开拓创新，改革开放和现代化建设取得新成绩，各项社会事业明显进步。

———坚持以工业化为战略核心，国民经济持续快速健康发展，经济结构战略性调整迈开新的步伐。预计全市完成国内生产总值１０９．１亿元，比上年增长１５．２％，增幅高于全省２．２个百分点。其中，一产增长２．５％，二产增长２３．３％，三产增长９．９％。全社会固定资产投资５２亿元，增长３３．３％；社会消费品零售总额３４．８亿元，增长１１．０％。实现财政总收入１４．５３亿元，其中地方财政收入６．２５亿元，分别增长３２．１％和２５．０％。财政总收入占国内生产总值的比重比上年提高１．１个百分点。三次产业结构发生可喜变化，第二产业增加值占国内生产总值的比重较上年提高４．２个百分点。全市规模以上工业增加值增长２７．５％，实现利税增长７７．０％，其中民营工业增加值、利税总额分别增长５８．０％和５７．０％。市属规模以上工业企业实现整体扭亏。工业经济效益综合指数较上年攀升１８个百分点。工业拉动经济增长９．０个百分点。金融支持经济发展力度加大，各项贷款增长创历史最高水平。经济发展后劲进一步增强。全市实施投资千万元以上工业技改项目２６个，９个重点项目已竣工投产；实施重点农业项目２３项，已建成国家级农业生产基地４个，建成省级无公害农产品基地１１个。农业产业化加速推进，恩达公司等５家企业跻身首批省级农业产业化重点龙头企业行列。

———坚持以城市建设为中心，基础设施建设进一步加强，城乡面貌发生新的可喜变化。着手编制了城市概念性形态规划，建成了城市规划展示馆。正式提出并着手组织实施了“两江四岸、组团开发”的城市建设总体构想。高新开发区新城区已基本完成以“三横八纵”为主的道路框架建设；仙来新区已完成总规、控规编制及行政中心建设整体招标等前期工作；仙女湖旅游码头、旅游公路建成使用，以干宝路、搜神路、仙女湖大道等三路建设和仙女湖行政中心为主的旅游城重点建设项目陆续开工，初步奠定了主城区“一城五区”的建设格局。继续实施城市绿化、美化、亮化工程，仙来大道“穿衣戴帽”示范工程圆满完成，我市被评为全国园林绿化先进城市。大力推进“一园二桥三路四岸五湖”建设，北环东路、高新大道、新欣互通立交建成通车，上新线景观大道、天工大道南段、渝州大桥建设进展顺利，成功引进外资独资兴建天工大桥及连接线工程；袁河综合整治一期工程已全面完成，仙来园、晚晴湖、百树园、百花湖建设相继完工。市污水处理厂一期工程、城市防洪三期工程等基础设施项目开工在建。一批现代化住宅小区拔地而起。副食品批发市场盛德商城等一？？？市场相继建成。村村通水泥路工程三年决战首战告捷。以河下小康红旗镇建设为重点，新一轮小城镇建设全面启动。

———坚持以大开放为主战略，加速推进体制机制创新，开放型经济发展跨入新的阶段。国企改革继续深化，流通企业两年改制攻坚拉开帷幕。分宜驱动桥厂成功实现整体出售，长林集团债转股工作和产权多元化改革取得新突破，前卫公司、通用机械厂等企业破产顺利实施。新一轮县乡机构改革全面完成，财政、金融体制改革稳步推进，分宜县新型农村合作医疗改革试点进展顺利，农村税费配套改革走在全省前列。全面推行经营性土地挂牌招标拍卖，城区基准地价平均增长３０％。大力推进招商创新，全市组建招商小分队１６０余支，聘请２１位知名人士担任招商顾问，积极组织和参与各类重大招商活动１２次，开放型经济发展取得明显成效。全市新签利用国外、境外资金合同项目２３个，新签利用国内市外资金项目１９０个。肯德基、华翔、飞宇、东亚鞋业等一批知名企业相继落户新余。预计全年实际利用国外境外资金３２００万美元，增长５．６％；实际引进国内市外资金３０亿元，增长１９．０％。

———坚持以人为本，人民生活水平进一步提高，社会保障事业得到新的发展。城镇居民人均可支配收入７０８７元，增长１０．６％。农民人均纯收入２８５０元，增加１３２元。城乡居民储蓄存款余额８１亿元，增长１８％。就业再就业各项工作任务全面完成。安置“４０５０”人员再就业１１００人，国有企业下岗职工再就业６５００人；实现省外劳务输出１６．１万人；城镇新增从业人员１．６４万人。采取调剂公益性就业岗位等办法，解决困难家庭子女就业５０人。“两个确保”得到加强，三条保障线向两条保障线平稳过渡，“四不欠”成果进一步巩固。共为３１１３３名离退休人员支付养老金１９４２２万元，发放下岗职工基本生活费１２９３万元，代缴三项社会保险费１３４６万元。社会救助体系进一步完善，城镇低保对象基本实现应保尽保，２８８７８名农村特困群众得到有效救助，建成了全省一流的收容救助站。

———坚持以经济社会统筹发展为战略方针，民主法制建设进一步加强，各项社会事业实现新的进步。科技项目申报工作取得明显成效，申报列入省级重点新产品计划项目１１项，列入国家级计划项目４个；４家企业列入省级以上高新技术企业。基础教育、高等教育稳步发展，农村教育网点布局调整更趋合理，民办教育、职业教育、信息技术和农村中小学卫星远程教育走在全省前列。农村有线广播电视村村通工程顺利实施，一批优秀广电、文艺作品在全国、全省获奖，成功举办了复市２０周年系列庆典活动。公共卫生长效机制得到加强，抗击“非典”斗争取得了疫情零发生的重大胜利。群众性体育活动蓬勃开展。五大上网工程全面实施，信息化工作跨入全省先进行列。以创建全国文明城市为主题，大力加强精神文明建设，市民素质得到普遍提高。计划生育、妇儿、信访、安全生产工作继续保持全省领先。积极开展创建平安市活动，第９次荣膺全省社会治安综合治理先进市。切实加强政府法制、行业纠风和反腐倡廉工作，加快建立公正透明的政务环境，政府依法行政水平进一步提高。加强基层民主建设，分宜县、渝水区分别被评为全省村民自治模范县（区）。？？管武装坚强有力，国防动员体系基本建立，人防和民兵预备役工作进一步加强，军政军民团结，连续四届荣获“全国双拥模范城”称号。金融、保险、工商、税务、供电、邮政、通讯、技术监督、烟草、盐业、医药、气象、地矿、新闻出版等各项事业实现新发展，环保、审计、统计、物价、人事、编制、外事、侨务、民族宗教、档案、老龄、残联、地方志、驻外机构等各项工作都有新的进步。

各位代表，２００３年的经济工作是在遭遇“非典”和严重干旱的困难局面下展开的。面对严峻挑战，全市上下万众一心，众志成城，坚持一手抓防“非”、抗旱工作不松劲，一手抓经济建设不动摇，在战胜“非典”和严重干旱等多种灾害的同时，国民经济和各项社会事业继续快速发展。成绩的取得来之不易！这是全市人民在省委、省政府和市委的正确领导下开拓创新、团结拼搏的结果，是人大依法监督、政协民主监督和积极参与支持的结果，是驻市中央省属单位、部队、武警、各民主党派、工商联、人民团体、离退休老同志和海内外友人大力支持的结果。在此，我代表市人民政府，向所有关心、支持新余发展的同志们、朋友们表示衷心的感谢和崇高的敬意！

同时，我们也清醒地看到发展中存在的困难和问题：支柱产业单一，非公有制经济发展相对滞后；城市建设精品不多，规划体系不健全，城市文化品位有待进一步提升；农民收入增长不快，城镇居民就业再就业压力加大；部分群众生活困难，社会不稳定因素依然存在。对此，我们正在采取有效措施，努力加以解决。

二、坚持“一条主线，五篇文章”的总体思路，加快建设现代化区域中心城市

市委五届五次全会作出了加快区域中心城市建设的重大决定，这是当前和今后一个时期我市经济、社会发展的中心任务，是贯彻落实“一条主线，五篇文章”总体思路的重大战略举措。面对新的形势和新的任务，各级政府务必始终坚持发展这个执政兴国的第一要务，与时俱进，开拓进取，以率先崛起的姿态和更加扎实有效的工作，加快现代化区域中心城市建设，努力开创各项工作的新局面。

按照市委、市政府确定的２００７年国内生产总值比２００２年翻一番、提前５年在全省率先实现全面小康的中长期战略任务，未来４年，我市国民经济要继续保持年均１５％以上的增长速度，到２００７年，力争全市国内生产总值达到２００亿元，人均国内生产总值达到１７０００元；财政总收入超过２７亿元，地方财政收入达到１２亿元，财政总收入占国内生产总值的比重由目前的１３．３％提高到１４．２％。城镇居民人均可支配收入、农民人均纯收入分别达到１１０００元和３４００元。工业主导型经济增长格局进一步巩固。高新开发区和工业园区成为拉动工业经济增长的主要载体。基本形成以钢铁为龙头、钢铁工业和其它工业齐头并进的多元支柱产业体系。现代服务业快速发展，物流、教育、旅游三大支柱鼎立发展的格局进一步确立，胜利路、城北广场等城市闹区改造全面实施，现代教育城、区域性物流中心、旅游休闲中心基本形成。以农业产业化为主导的农业和农村经济发展格局进一步强化。对接长珠闽、融入全球化进程明显加快，利用外资成为拉动投资增长的主渠道。各项改革全面深化，国有工商企业改组改制全面完成，以非公有制经济为主体、股份制改造为基本模式的多元产权结构初步确立，率先在全省建立比较完善的社会主义市场经济体制。城市化速率不断加快，城市文化品位不断提升，区域中心城市地位进一步确立。高新开发区新城区、仙女湖旅游城、仙来新区基本建成，“百街改造”和旧城改造全面实施，抱石公园、体育中心、新人民医院、人才劳动力中心和城区中心的文化商业广场等一批精品工程建设相继完成，主城区建成区面积达到４８平方公里。城乡基础设施和功能日趋完善，以分宜镇、罗坊镇等１０个中心镇为烘托的城镇体系不断健全，城市化速率达到５０％以上，基本实现城市化。

今年是加快建设全面小康社会的关键之年。根据中央经济工作会议和省委第十一届四次全会精神，全市经济和社会发展的总体要求是：以邓小平理论和“三个代表”重要思想为指导，全面贯彻党的十六大和十六届三中全会精神，坚持“一条主线、五篇文章”的总体思路，以经济结构调整为重点，以扩大开放为主动力，大力实施项目推动战略，坚定不移地加速工业化、城市化和农业产业化，不断增强城市的总体实力和综合竞争能力。树立全面、协调、可持续的科学发展观，认真落实“五统筹”的要求，科学安排好各项工作，实现国民经济持续快速协调健康发展和社会全面进步。

经济、社会发展的预期目标是：国内生产总值１２５．６亿元，比上年增长１５％；全社会固定资产投资６７．６亿元，增长３０％；社会消费品零售总额３８．８５亿元，增长１１．５％；财政总收入１６．７亿元，其中地方财政收入７亿元，分别增长１５％和１２％；城镇居民人均可支配收入增长１０％，农民人均纯收入增加１２０元；城镇登记失业率控制在３．５％以下；人口自然增长率控制在省定目标之内。

加快建设现代化区域中心城市，要牢固确立以城市为中心带动经济社会发展的指导思想，做强城市产业，做大城市规模，提升城市品位，不断提高城市经营管理水平，着力打造独具魅力的城市品牌，努力把新余建成赣西地区最适宜人居注最适宜人创业的现代化中心城市，加速产业集聚、要素集聚“洼地效应”的形成。

加快建设现代化区域中心城市，要切实加大体制机制创新力度，大力发展非公有制经济，加快建立现代企业制度，完善市场体系，大胆破除一切影响发展的体制弊端，放手让一切加快发展的活力竞相迸发。坚持从更高层面上扩大对外开放，面向沿海发达地区形成更加开放的接纳态势，全方位拓展中心城市的发展空间。

加快建设现代化区域中心城市，要继续坚持城市经营与产业发展的良性互动，把做大做强优势产业作为提升城市核心竞争力的关键。大力实施品牌城市战略，加速引进和着力打造一批品牌企业、品牌产品。积极探索新型工业化道路，加快高新技术产业化和传统产业现代化改造；大力发展以物流为重点的现代服务业，加快发展现代农业，着力壮大技术先进、集中度高、关联密切、特色鲜明的优势产业集群，不断增强城市的产业支撑。

加快建设现代化区域中心城市，要始终坚持以人为本和全面、协调、可持续的科学发展观。统筹城乡发展，统？？？区域发展，统筹经济社会发展，统筹人与自然和谐发展，统筹对外开放与对内发展。始终把增加城乡居民收入、改善人民生活作为政府工作的出发点和落脚点，正确处理改革、发展、稳定的关系，切实做好就业再就业工作，加快构建城乡一体的就业和社会保障体系，努力使人民群众从改革发展中得到更多的实惠。紧紧依靠全市人民，把改革开放和现代化建设事业发展好。

三、坚定不移地实施大开放主战略，全面加速体制机制创新

坚持把招商引资作为经济工作的生命线，大力发展开放型经济。加速推进招商主体、招商方式和招商机制的创新，积极探索专业化、市场化、社会化招商的有效途径，着力加强招商小分队建设，积极聘请一批中介机构、国内外知名企业、社会知名人士和沿海地区离退休老干部开展委托招商。制定更加优惠的政策，鼓励招商致富，鼓励民间团体和市内外人员利用自身优势参与招商，鼓励市内骨干企业依托产业、资源、市场等比较优势展开招商，加速形成以企业为主体的招商新机制。大力实施品牌依附战略，跟踪抓好与美国华新顿、香港地洲、上海复星、福建神龙、江锂新材料公司、张扬集团等知名企业的合作洽谈。切实加强项目库建设，积极推行项目委托、项目采购制度，面向专门机构、专业人才和全社会公开征集项目，加速形成项目滚动储备、开发的新机制，突出抓好一批对国民经济和社会发展有重大影响的关键性项目。用足用好国家有关政策，积极争取更多的国债项目、“双高一优”项目。实施市场多元化战略，加快培育一批出口创汇型骨干企业。确保全年实际利用国外境外资金３５００万美元，实际引进国内市外资金３５亿元；完成外贸出口２０００万美元。

坚持把民营经济发展作为加快新余经济发展的重大战略举措，放手让一切创造社会财富的源泉充分涌流。进一步放宽市场准入，全面落实民营企业的国民待遇。积极鼓励下岗职工创办个体私营企业，鼓励外出务工经商人员回乡投资兴业，鼓励科技人员离职、兼职创办民营企业。支持民营企业嫁接改造国有、集体企业，全面推进国有工商企业改组改制，加快完善企业法人治理结构。充分发挥企业家协会等中介组织的作用，完善中小企业担保中心职能，积极帮助民营企业解决融资、技术服务等问题。突出抓好省级民营科技园建设，支持一批重点民营科技企业做大做强。鼓励和引导民营企业实施靠大联强战略，加快组建民营企业集团。

积极开展“全民安商年”活动，着力营建扩大开放的环境平台。坚持把构建“信用新余”与创建投资、金融安全效益区结合起来，以精减行政审批事项、加快政务超市建设为重点，全面优化政务环境；以建立健全企业信用等级评定制度、扩大信用放贷为重点，加快建立良好的信用环境；以提高项目运作时效为重点，全面提高服务水平和服务质量；以加快海关、商检机构和公路网络建设为重点，进一步完善对外开放条件，全力打造新余开明开放的形象品牌。全面推行重大项目代办制、领导跟踪服务制和落实情况督查制，积极推进行政审批制度改革。加大投资环境专项整治力度，严肃查处侵害投资者权益的违法行为。

四、坚定不移地推进城市化，在加快组团式城市开发中着力打造独具魅力的城市品牌

大力推进三大功能新区建设，加快形成“五位一体”的城市格局。精心组织城市总规修编，抓紧制定城市分区规划，配套搞好“两江四岸”和城区主干道控制性、修建性详规，严格按规划组织新城开发。以商务大楼、时代广场建设为重点，大力推进高新开发区新城起步区建设，加快管网设施进区步伐，积极启动南部片区五条主干道建设。加快兴建仙女湖旅游城，年内建成仙女湖行政中心，实现三路通车。抓紧天工大桥及连接线等重点项目建设，确保渝州大桥按期完工。实行整体招商、整体建设、整体交付的方式，全面启动仙来新区一期工程建设，加快渝水区行政中心整体搬迁。进一步完善主城区路网体系等基础设施。开工新建中山路、长青南路等一批城市道路，抓紧长青南路立交桥建设，全面完成北湖东路等道路续建。继续抓好城市防洪工程、供水管线铺设、污水处理厂一期工程和生活垃圾填埋场等市政公用设施建设。

精心组织实施城市立体绿化、美化、亮化工程，高标准创建国家级园林城市。切实抓好城市大环境绿化，大力发展城郊水源涵养林和环城绿化带，积极启动高新开发区、仙女湖旅游城大生态系统建设，加速推进浙赣铁路防护林、城西绿化隔离带、“两江四岸”绿化带建设。实施拆围透绿，高标准组织单位庭院绿化美化整体改造。注重造型设计，着力打造一批标志性绿化景观。继续推进“两江五湖”治理，高起点实施抱石公园改造。全力组织实施管线下地工程。做好２００５年大规模改造城区街道前期准备工作，全面推进城区主干道“穿衣戴帽”工程。积极启动环城文化走廊建设，高水准建成仙来东大道、校园路、清宜公路西段等标志性城市进出口景观。实施精品工程，着力抓好市体育广场艺术中心等一批精品建筑，加快建设一批现代化住宅小区。

创新城市经营理念，不断提升城市经营管理水平。把土地经营作为城市经营的核心。垄断一级市场，强化土地储备，高度集中城市土地的供应权。实行计划调控供地、规范适度投放，通过创造城市闹区、完善基础设施、强化增殖预期等手段，不断涵养提升地租级差。高度重视发展房地产业，努力激活房地产市场实施鼓励住房消费的积极政策，鼓励农民和市外居民进城落户，鼓励市民住房以小换大、以旧换新，搞活二级市场；加强市场调控，加快建立满足多层次、多样化需求的房产供应市场体系。严格按规定征收各项规费，不断增强城市经营和城市开发的发展后劲。加速推进城市资源整合，解决好企业办城建问题，使城市的水、电、气、房地产和园林绿化等各项事业纳入政府的统一规划、统一建设、统一管理。积极推进城市供水、供气、公交等事业单位股份制改造。组建管线沟公司，加速实现管网设施所有权、经营权、使用权相分离。按照“花钱买管理、不花钱养人”和管、保分离的原则，公开拍卖城区道路保洁权和园林、市政养护作业权。以完善城管“１１０”为重点，全面提升城市管理服务水平。积极组织开展创建全国社区建设先进市活动，理顺社区管理体制，强化社区服务职能，加快现代文明社区建设，争取用两年时间，建立完善以社区为主体，市、街道、居委会和社区四级联动的城市管理新体制。加快城中村改造步伐，有序推进旧城整体改造开发。对城中村农民建房，坚持统一规划、集中审批、先批后建的原则，由市政府每季度审批一次，下决心解决违章建筑问题。实施畅通工程，规范营运市场，坚决取缔摩托车等非营运车辆营运行为，规划、建设好停车场和城市立交设施，实现城市交通秩序的明显改善。

五、坚定不移地推进工业化，高起点构建中心城市的产业支撑

把支持中央、省属驻市企业作为加快新余工业发展的重大战略举措，全力支持驻市企业加快发展。主动承担企业办社会职能，积极协助驻市企业调整结构、壮大主业。加快铁路二通道建设。协助抓好江西二化依法破产和江西钢丝厂债转股工作，积极促成江西锂厂、江西锻压厂、分宜煤矿电机厂等企业改制。突出钢铁产业的链式化发展，以新项目、新工艺、新产品为方向，深度开发钢铁后续加工、上下游产品及相关配套产业，大力引进和发展五金、电器等相关企业，加速形成一批以钢铁为原材料及其配套的产品集群，高起点培育壮大南方钢铁精品基地。

积极探索新型工业化道路，加快用高新技术改造提升传统产业。继续抓好新钢中厚板、国电公司５００千伏输变电站、飞宇电声工业园等１４个续建项目，积极组织实施新钢转炉二期工程、７号高炉大修改造、新余华源喷气织机生产线、良山钢管改扩建、分宜电厂２０万千瓦循环硫化床机组技改、城北２２０千伏输变电工程等一批新开工项目。鼓励支持新余制药、新和药业、欧氏药业等一批医药企业加快发展，积极推进中药现代化生产，加快中药材基地建设，推动医药产业规模化、链式化发展。

着力组织实施工业“三年翻番”战略，力争全面实现市属规模以上工业“三年翻番”目标。积极帮助重点骨干企业跑项目、争资金，争取促成分宜海螺新增日产５０００吨水泥等项目的立项开工。继续实施靠大联大、扶优扶强策略，抓紧长林集团、江西电工厂、床上用品厂等企业改组改制工作，大力引进和发展产业关联度高、综合竞争能力强的优势企业，重点扶持华源、天翔、飞宇、海螺等一批骨干企业做大做强，加快培育形成多元支柱产业。

大力推进工业园区建设，举全市之力加快高新开发区发展。积极推进开发区管理主体与经营主体相分离，组建具有法人资格的开发经营公司具体从事开发区经营，鼓励境内外有实力的经营实体参与经营园区开发，鼓励开发区现有企业和投资开发商以园区为单位招商引资，千方百计引进一批重大骨干项目和知名企业进区落户。加紧制定配套完善的鼓励政策，积极引导新办工业项目和城区工业企业向开发区集中，鼓励新钢、新电、赣西供电局等骨干企业利用自身优势创办特色工业园区。努力把开发区建成我市经济增长极、对外开放窗口、劳动力就业基地和现代化城市新区。坚持将工业园区建设作为壮大县域经济的主要载体，加速分宜城西工业园、分宜夏布城和资源开发型工业重镇建设，鼓励有条件的地方发展特色产业园，以园区建设构筑工业快速发展的新高地。

切实加强企业家队伍建设，进一步形成尊重企业家、爱护企业家、支持企业家发展的良好氛围。积极探索股权期权等多种分配形式，健全企业经营者激励约束机制。大力引进和培育企业高级管理人才、职业经理和技术创新带头人，加快完善市场化选聘企业经营管理者机制。积极引导民营企业加快建立现代企业制度，推动？？？营企业从家族式管理向现代化公司制企业管理转变。

六、坚定不移地推进农业产业化和农村城镇化，努力增加农民收入

加快发展农业产业化，着力培育全国、全省知名的特色农业基地。坚持以基地开发促进产业开发，以龙头企业培育带动产业规模发展。大力引进一批产业化知名龙头及配套企业，鼓励扶持现有龙头企业做大做强。继续抓好现代农业示范园区建设，积极启动生态养殖工程，高标准建设１００个生态养殖小区。依托福润万吨肉制品深加工、恩达１００万套夏布床上用品生产线等产业化项目实施，加快５万亩花卉苗木、６万亩苎麻、１２万亩棉花、１０万头肉牛、５０万头生态养猪、５万亩新余蜜桔、香椿丰产林等基地开发。大力加强产业带中心乡镇建设，加速形成“一县一业、一乡一品”的发展格局，促进花卉苗木、草食畜禽、优质棉麻、绿色果蔬等特色农业产业的专业化生产、区域化布局。鼓励土地合理有偿流转，引导农业生产要素向龙头企业和种养大户加速集中。

加强农产品市场建设，全面提升农副产品市场竞争力。积极开展“三绿工程”、“农改超”试点工作，加快农副产品绿色市场建设，高起点建成分宜苎麻大市场大力发展“订单农业”， 加快发展各种专业协会和中介组织，积极开辟面向沿海的农产品销售通道。推广良种良法，加强农业科普示范基地建设，加速培育仙女湖、卫岗、恩达、百乐等一批知名农业品牌。继续推进信息网络村村通工程，加紧组建市、县（区）两级质检机构，健全动物重大疫病预防和控制机制。

大力推进小城镇建设，着力改善农村生产生活条件。坚持以科学规划为先导，突出特色，创新机制，高标准抓好分宜镇、罗坊镇等１０个中心镇建设。重点搞好分宜县城东新区开发，抓紧实施县行政中心整体搬迁。强化城镇管理，优化人居环境，吸引农民加速向小城镇集聚。切实加强新余至周边县市的干线建设，加速形成由主城区到达周边县城的一小时交通路网。加紧建成由主城区、分宜县城至各乡镇的半小时公路交通体系，确保按期完成农村公路三年全面改造任务。完善规划设计，美化居住环境，积极推进以小康红旗村为重点的新农村建设。高度重视和加强农田水利基本建设，继续推进农村电网改造，重点抓好袁惠渠续建配套工程和彰湖、鹄山水库及重点小（一）型水库除险加固与节水改造项目。加强森林资源管理，全面完成退耕还林工程，大力组织实施长江上中游江西造林工程、农村沼气建设等生态项目。继续抓好江口库区移民工作。加快中低产田改造和园田化建设，切实保护基本农田，提高粮食综合生产能力。继续深化农村税费配套改革，坚决防止农民负担反弹。

七、加速推进商贸物流中心、旅游休闲中心和现代教育城建设，大力发展现代服务业

加快商贸体系建设，着力打造赣西最大的现代物流中心。按照“城郊大市尝城内大商潮的要求，进一步完善中心城区商贸体系和网点布局，加速新世纪购物广场商贸城、圣德购物广场步行街、金马购物广场等项目兴建，抓紧规划建设新余家电大世界、嘉汇购物广场等一批市常创造城市闹区，积极推进胜利北路商业繁华街改造，精心组织实施城北广场人民防空工程，高品位建成城？？？、城北两个多功能、综合性商业文化广场加快实施流程再造工程，大力发展第三方物流。突出抓好高新开发区物流带兴建，加快铁路货运中心、公路货运中心建设，抓紧组建以货运配载为纽带，融仓储、配送、电子商务、多式联运等多功能于一体的大型物流中心，发展集装箱运输，加快建设赣西的陆地港口。

做大做强旅游产业，着力打造高品位旅游休闲中心。切实抓好仙女湖旅游城建设，加快仙女湖、钤阳湖环湖旅游公路和旅游宾馆、同益别墅区等基础设施建设。积极争取促成以情山爱水为主题的仙女湖综合开发，加快建设凤凰湾休闲度假中心、舞龙湖大型水上娱乐中心、仙女湖生态游乐中心、九龙湾休闲游乐园、仙女湖狩猎场等一批旅游休闲项目，着手规划论证天工开物城、东方丽人城等主题公园建设。实行统一品牌策略，加大旅游产品开发和景区宣传推介力度，打捆做响仙女湖品牌。搞好旅游景点串线工作。提升景区接待条件和服务水平，努力把仙女湖景区建成全省知名的会议会展中心。策应仙女湖景区开发，大力发展工业旅游、生态旅游和城市观光等旅游项目。

发展大文化产业，着力打造现代知名教育城。启动现代职业教育基地建设，切实加强与国内外知名企业和重点院校的合作办学，抓紧中山学院、冶金学院、天工学院、远方学校、旅游酒店管理学校等院校建设，鼓励和支持渝州、赣西等骨干院校做大做强。发展少儿文艺特色教育，积极启动少儿文化城建设，加快组建一批高档次、全封闭特教学校。抓紧开工建设市体育中心，高水准策划和经营好第十二届省运会。加强文艺团体建设，高标准建设文化广场积极启动有线电视数字化改造，加快广播电视业发展。着力抓好市人民医院、中医院兴建改造，开办远程医疗，加快发展特色专科医院。

积极发展金融、保险、证券、信托业，完善金融市场体系，大力推进农村信用社改革，加快组建市农村合作银行。切实加快物业管理、家政服务、中介服务等社区服务业发展。着力培育会计、审计、评估、法律服务、职业介绍和建设监理等中介服务业市场，加快构建与国际接轨的中介服务体系。

八、建设城乡一体的就业和社会保障体系，统筹推进各项社会事业发展

坚持把就业再就业工作摆在关系国计民生的重大战略地位，加快构建覆盖城乡的就业体系。实施增加就业岗位工程，加快再就业基地和非正规就业劳动组织建设，加快中小企业和高就业型产业发展，积极开发社区服务和公益性就业岗位，鼓励提倡以多种形式和灵活方式实现就业。组织开展“再就业心连心”帮扶活动，积极举办就业招聘洽谈大会和“４０５０”人员专场招聘会，加大岗位调剂力度，重点帮助下岗失业人员、“４０５０”人员和困难人群实现再就业。实施积极的就业政策，放宽《再就业优惠证》发放范围，强化就业培训、小额贷款、税收优惠等多方面服务，鼓励下岗失业人员自主创业、自谋职业，鼓励用工单位吸纳更多的下岗人员就业。抓紧开工建设就业大市场，加快建立覆盖城市社区和各乡镇的就业信息网络和劳动保障服务体系，加大农民外出务工组织力度，积极开辟就业再就业“绿色通道”，争取用两年左右的时间，把新余建成全省知名的技能型劳务输出基地。规范劳动力市场秩序和企业？？工行为，切实保障劳动者合法权益。力争全年新增城镇就业１．６万人，下岗职工再就业６５００人，安置“４０５０”人员再就业１１００人，省外劳务输出１６．５万人。

继续巩固“两个确保”和“四不欠”成果，加快建立城乡一体的社会保障体系。完善以养老保险、最低生活保障为重点的保障制度建设，稳步推进下岗职工基本生活保障向失业保险并轨，妥善安置出中心下岗职工就业和生活保障工作，千方百计确保下岗在中心职工基本生活费和企业离退休人员养老金按时足额发放。做好改制企业职工社会保障关系接续工作，扩大社会保障覆盖面，将非公有制经济从业人员养老、医疗、失业保险等统一纳入社会保障体系建设。加强保费征缴，多渠道筹措社会保险资金。继续做好城市低保工作，积极探索建立农村基本医疗保险、养老保险、最低生活保障和孤寡老人社会赡养制度。建立全市统一的社会化管理服务网络，逐步将企业离退休人员移交社区管理。

坚持经济社会统筹发展的战略方针，全面加快各项社会事业发展。深化教育体制、办学模式、教学手段的改革，加速构建现代国民教育体系。加快高专专升本步伐，抓好高专和市一中新校区建设，加紧筹建青少年素质教育千亩实践基地和科普示范城，尽快建立面向名校、覆盖全市、具有远程教育功能的高速传输信息网络系统。整合教育资源，调整优化中小学网点布局。实施名师名校工程，加速引进和培育高素质的教学研人才，鼓励连锁办学和集团办学，加快建设一批品牌学校。开好市第五届运动会，精心举办建国５５周年系列文艺演出和纪念傅抱石诞辰１００周年系列活动。实施精品战略，加快文艺事业发展，努力打造具有新余特色的文化品牌。改善农村医疗卫生条件，全面加快新型农民健康保障机制建设。加紧市传染病医院、疾控中心、紧急医疗救援中心建设，建立健全公共卫生四大体系，着力提高突发公共卫生事件应急处置能力，坚决防止重大传染病和突发公共卫生事件的发生。实施可持续发展战略。加强农村土地整理，加快组建市土地整理中心。抓好仙女湖水源涵养地及生物多样性功能保护区和分宜县国家级生态示范区建设，积极启动城市环境保护十大工程。重视妇儿、老龄工作，加大妇女纲要、儿童纲要实施力度。深化人口与计划生育工作综合改革，以稳定低生育水平、提高人民群众满意率为目标，加快建立“依法管理、村（居）民自治、优质服务、政策推动、综合治理”的工作新机制。关心、支持国防和军队建设，深入贯彻国防法规，深化全民国防教育，完善国防动员机制，推进“双拥”共建。认真组织编制第十一个五年规划。

大力推进平安市创建工作，营造安居乐业的社会环境。把创建平安新余作为重要的城市品牌来抓，切实加强社会治安综合治理，强化普法教育，全面实施社区警务战略，深入开展“严打”整治斗争，完善社会治安防控体系。做好“法轮功”人员教育转化工作。高度重视群众来信来访，解决群众关注的热点难点问题。建立健全各种预警和应急机制，提高政府应对突发事件和风险的能力。完善安全生产监管体系，建立安全生产长效运行机制，深入开展安全生产专项整治，抓好消防安全、森林防火等工作，坚决杜绝重特大事故的发生。

九、加强政府自身建设，加快构建学习型、服务型、创新型政府

深化行政管理体制改革，合理调整各级政府机构设置，精简政府职能，理顺职能分工，切实把政府经济管理职能转变到主要为市场主体服务和创造良好发展环境上来。发展电子政务，实行政务公开，全面推行服务承诺制，加快实现由管理型政府向服务型政府转变。全面推进事业单位人事制度改革。坚持尊重劳动、尊重知识、尊重人才、尊重创造的方针，大力实施人才战略，加紧引进和培养现代化建设急需的人才，完善人才分类管理体制，改进人才培养方式，加快人才结构调整，优化人才资源配置，建立健全有利于人才脱颖而出、有利于人才充分施展才能的选拔任用机制，加快建设高素质的公务员队伍，为加快经济社会发展提供有效的人才支撑。

坚持依法治市、依法行政，改革行政执法体制，推进城市综合执法制度改革。积极办理人大代表、政协委员的提案和建议，认真听取民主党派、工商联、无党派人士及工青妇等人民团体的意见，充分发挥人民群众和新闻媒体的监督作用，拓宽反映社情民意的渠道，自觉把政府的行政行为置身于人大依法监督、政协民主监督和社会舆论监督之下。扩大基层民主，完善村（居）民自治。切实抓好领导干部廉洁自律、查办大案要案、纠正部门和行业不正之风三项工作，坚持从源头上、制度上预防和治理腐败。加强审计监督和廉政监察，强化领导干部任期经济责任审计，实行领导干部述职述廉制度。积极推行综合预算、部门预算和政府采购，全面实行国库集中收付。合理界定各级政府的财政支出责任，从严控制财政支出范围，加快构建公共财政框架。加强有形建筑市场建设，建立健全统一的房地产交易市常组建政务投诉中心，积极开展政务环境评议评价活动。努力建设高效廉洁政府。

加强思想作风和政府效能建设，不断提高政府学习、创新的能力。各级领导干部和政府公务人员要始终坚持立党为公、执政为民的根本宗旨，深入基层，贴近群众，关注民生，始终保持与人民群众的血肉联系，切实关心和解决人民群众的实际问题。坚持重实干、做实事、求实效，切实把精力转移到抓大事、谋大局、多做具体实际的工作上来。适应新形势，研究新情况，解决新问题，加强理论学习和知识更新，积极探索发展社会主义市场经济的有效途径，改进工作方法，加快制度创新，不断提高驾驭全局、解决实际问题的能力和水平。

各位代表！加快建设现代化区域中心城市，不断开创全面建设小康社会的新局面，是实现新余率先崛起的重大战略举措，是时代赋予我们光荣而神圣的历史使命。让我们紧密团结在以胡锦涛为总书记的党中央周围，在省委、省政府和市委的领导下，深入贯彻党的十六届三中全会和省委第十一届四次全会精神，解放思想，开拓创新，励精图治，团结拼搏，全面完成今年的各项目标任务，为加快建设全面小康社会做出新的贡献！
